# Analysis of admixed Greenlandic siblings shows that the mean genotypic values for metabolic phenotypes differ between Inuit and Europeans

**DOI:** 10.1186/s13073-024-01326-3

**Published:** 2024-05-23

**Authors:** Long Lin, Mette K. Andersen, Frederik Filip Stæger, Zilong Li, Kristian Hanghøj, Allan Linneberg, Niels Grarup, Marit Eika Jørgensen, Torben Hansen, Ida Moltke, Anders Albrechtsen

**Affiliations:** 1 Section for Computational and RNA Biology, Department of Biology, University of Copenhagen, Ole Maaløes Vej 5, 2200 Copenhagen, Denmark; 2 Novo Nordisk Foundation Center for Basic Metabolic Research, Faculty of Health and Medical Sciences, University of Copenhagen, Blegdamsvej 3B, 2200 Copenhagen, Denmark; 3 Centre for Clinical Research and Prevention, Bispebjerg and Frederiksberg Hospital, The Capital Region of Denmark, Copenhagen, Denmark; 4 Department of Clinical Medicine, Faculty of Health and Medical Sciences, University of Copenhagen, Copenhagen, Denmark; 5 Centre for Public Health in Greenland, National Institute of Public Health, University of Southern Denmark, Copenhagen, Denmark; 6 Clinical Research, Copenhagen University Hospital – Steno Diabetes Center Copenhagen, Herlev, Denmark; 7 Steno Diabetes Center Greenland, Nuuk, Greenland

**Keywords:** Genotypic value, Greenlandic population, Admixture, Sibling analysis

## Abstract

**Background:**

Disease prevalence and mean phenotype values differ between many populations, including Inuit and Europeans. Whether these differences are partly explained by genetic differences or solely due to differences in environmental exposures is still unknown, because estimates of the genetic contribution to these means, which we will here refer to as mean genotypic values, are easily confounded, and because studies across genetically diverse populations are lacking.

**Methods:**

Leveraging the unique genetic properties of the small, admixed and historically isolated Greenlandic population, we estimated the differences in mean genotypic value between Inuit and European genetic ancestry using an admixed sibling design. Analyses were performed across 26 metabolic phenotypes, in 1474 admixed sibling pairs present in a cohort of 5996 Greenlanders.

**Results:**

After FDR correction for multiple testing, we found significantly lower mean genotypic values in Inuit genetic ancestry compared to European genetic ancestry for body weight (effect size per percentage of Inuit genetic ancestry (se), −0.51 (0.16) kg/%), body mass index (−0.20 (0.06) kg/m^2^/%), fat percentage (−0.38 (0.13) %/%), waist circumference (−0.42 (0.16) cm/%), hip circumference (−0.38 (0.11) cm/%) and fasting serum insulin levels (−1.07 (0.51) pmol/l/%). The direction of the effects was consistent with the observed mean phenotype differences between Inuit and European genetic ancestry. No difference in mean genotypic value was observed for height, markers of glucose homeostasis, or circulating lipid levels.

**Conclusions:**

We show that mean genotypic values for some metabolic phenotypes differ between two human populations using a method not easily confounded by possible differences in environmental exposures. Our study illustrates the importance of performing genetic studies in diverse populations.

**Supplementary Information:**

The online version contains supplementary material available at 10.1186/s13073-024-01326-3.

## Background

**Fig. 1:**
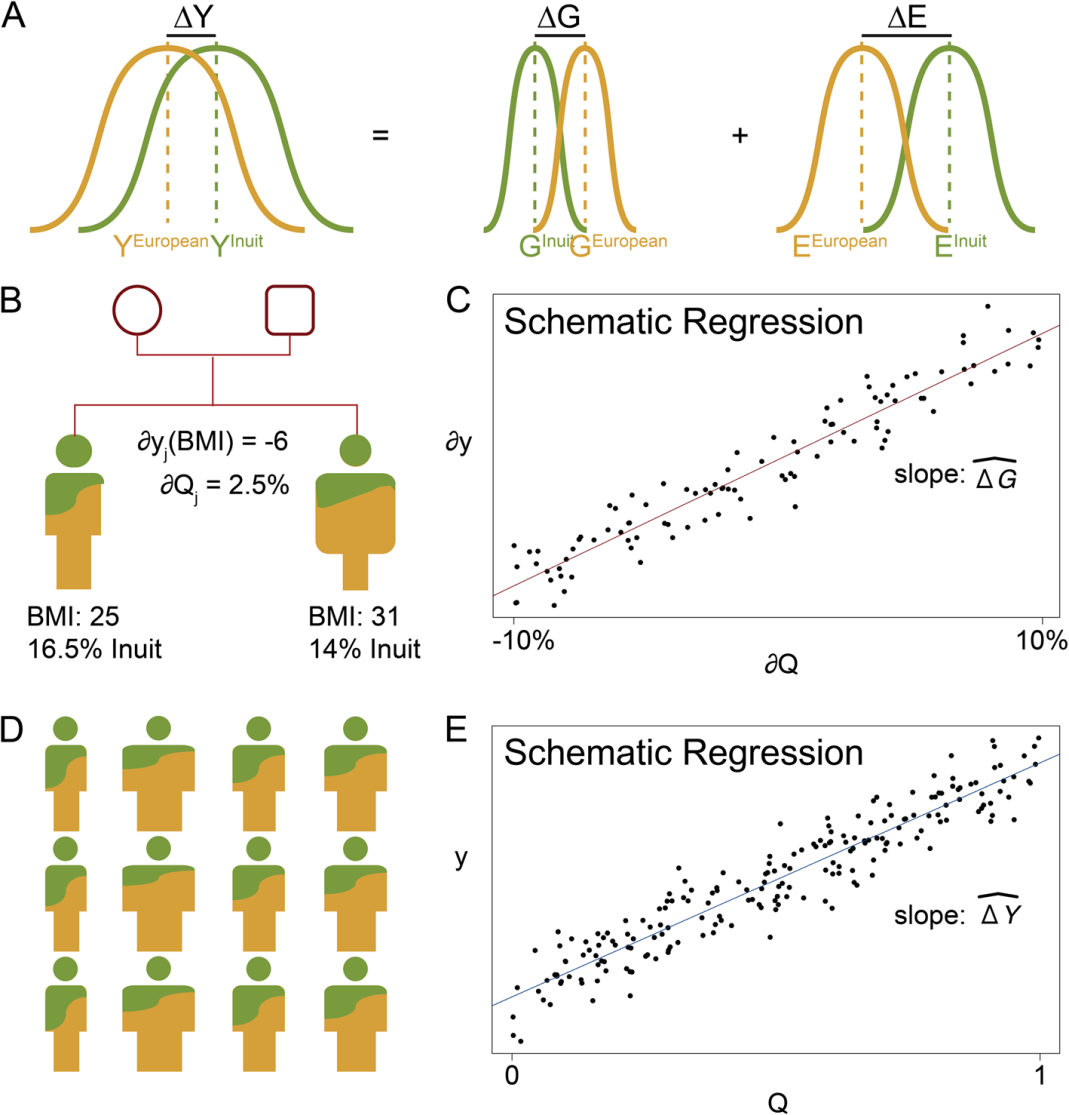
Illustration of the study design with body mass index as an example phenotype. **A** An illustration of the concept that the difference of mean phenotypic values (∆Y) is composed of the difference in mean genotypic values (∆G) and the difference in mean environmental effect (∆E). **B** A hypothetical example of a pair of admixed full siblings with both Inuit genetic ancestry (green) and European genetic ancestry (yellow). In the example they have −6 difference in body mass index (BMI) and 1.5% difference in Inuit ancestry proportion. **C** Regression of difference in phenotypic values (*∂y*) with difference in ancestry proportions (*∂Q*) within pairs of admixed full siblings based on 100 pairs. The slope is an estimate of the difference in mean genotypic value (∆G) between Inuit and European genetic ancestry. **D** A hypothetical example with 12 admixed individuals with different values of BMI and different amounts of Inuit genetic ancestry. **E** Regression of phenotypic values (*y*) with ancestry proportions (*Q*) based on 200 admixed individuals. The slope is an estimate of the difference in the mean phenotype value (∆Y) between Inuit and Europeans

Phenotypic difference between human populations is mainly driven by difference in environmental exposures, even when the phenotype has a high heritability [1]. However, the difference may also partly be due to differences in the genetic contributions to the phenotype (Fig. 1A). The genetic contribution is sometimes called the genetic load, the polygenic effect, or the genotypic value of a given phenotype [2]. Here we use the term genotypic value (G), defined as the sum of effects of every causal genetic marker for a given phenotype. Different populations can in principle have different mean genotypic values for a phenotype, which is important for understanding relative disease prevalence, heritability and fitness in evolution in different populations. However, it is not straightforward to estimate the difference in mean genotypic values between populations (∆G). In particular, it is problematic to do it by estimating the mean phenotypic value for each of the populations and then comparing the estimates, as these estimates of mean phenotypic values are easily confounded by environmental exposures which often differ between the populations. Currently, the mean genotypic value of a given population cannot be estimated directly, because not all causal variants and their effect sizes for a given phenotype are known. With the availability of large-scale genome-wide association studies, polygenic scores (PGS) can be used as a way to estimate mean genotypic values [2], but the performance of a PGS will vary in different populations and, thus, confound the estimates of the mean genotypic values [3]. This is particularly a problem when there is a large genetic distance between the population in which the PGS is trained, and the population in which the mean genotypic value is estimated [3].

To overcome these challenges, we here leverage the nature of genomes from the recently admixed Greenlandic population. Admixed genomes are mosaics of different ancestral segments [4], and for each admixed individual, it is possible to accurately estimate the proportion of genetic ancestry derived from each ancestral population [5] as long as the ancestral populations are sufficiently genetically distinct. The majority of the genetic ancestry of present-day Greenlanders is Inuit [6]; however, in recent time, there has been a large amount of gene flow from Europe, mainly due to the Danish colonisation [7]. There is a large variation in the proportion of Inuit and European genetic ancestry across the Greenlandic population, but on average they have 25% European and 75% Inuit genetic ancestry [6] and, importantly, Inuit and European genetic ancestry differ enough for estimation of ancestry proportions to be accurate [6, 7].

Notably, genetic ancestry is correlated with environmental influence, including socio-economic status [8] and sociocultural practices [9], which confounds the estimation of differences in mean genotypic values; however, we alleviate this environmental confounding by restricting the analysis to admixed full siblings. Such siblings have equal probability of inheriting any given allele from each parent and are expected to have similar environmental exposures [10]. Thus, the expected genetic ancestry of both siblings is the mean genetic ancestry of their parents. However, due to random recombination, there will be small differences in ancestry proportion between siblings, and importantly, this variability is independent of environmental exposures [11]. This is important, because under the assumption that the difference in the ancestry proportions of full siblings is independent of the difference in their environmental exposures, it is possible to estimate the difference in mean genotypic value (∆G) between two genetic ancestral populations, like Inuit and European ancestry, by estimating the slope of the line obtained when regressing the difference in phenotypic values (*∂y*) with the difference in ancestry proportions (*∂Q*) between full-sibling pairs that have genetic ancestry from both populations (Fig. 1B,C). Similarly, we can also estimate the difference in mean phenotypic value (∆Y) between the two ancestral populations from individuals with genetic ancestry from both populations using a regression slope estimate (Fig. 1D, E). Notably, the difference in mean phenotypic value will be affected by environmental differences between populations (Fig. 1A), whereas the difference in mean genotypic value will not, since the genotypic value per definition solely describes the genetic contribution to the mean phenotype of a population. And importantly, when using the described design to estimate the difference in mean genotypic values, the estimates are not easily confounded, which we will show via simulations in this paper. We note that this design is similar to the PGS-based sibling design proposed by Selzam et al. [8], but with the key difference that the design presented here is based on ancestry proportions from an admixed population instead of PGS.

The demographic history of the Inuit ancestors of the Greenlanders differs from many other populations by having recently undergone a long population size bottleneck. This has led to a larger variance in the genotypic values of phenotypes despite not having a large effect on the overall genetic load of fitness [12]. This means that mean genotypic value in the Inuit is not expected to on average be higher or lower for a given disease or phenotype. But for specific diseases or phenotypes it is plausible that genetics explain part of the mean phenotypic differences observed in Inuit compared to other populations and thus that mean genotypic values for these specific diseases or phenotypes differ. This would be in line with previous observations of genetic variants unique to the Inuit, which have substantial impact on metabolic phenotypes, including height, body weight, type 2 diabetes, HbA1c, circulating low-density lipoprotein (LDL) cholesterol and fatty acids [13–18], making this a particularly relevant population to study in the context of mean genotypic value differences.

Therefore, we aimed to investigate if genetics explains parts of the differences in mean phenotype values between Inuit and Europeans by estimating the difference in mean genotypic values based on differences in ancestry proportions between admixed full siblings identified in a cohort of 5996 Greenlanders.

## Methods

### Study participants

We included Greenlanders from the population health surveys B99 (sample size 1953, recruited 1998–2001) [19], Inuit Health in Transition (IHIT; sample size 2807, recruited 2005–2010) [20] and B2018 (sample size 1236, recruited 2017–2019) [21]. All these cohorts were collected as part of a general population health survey of the adult (18+ years of age) Greenlandic population based on random population samples. Genomic data was utilized to estimate ancestry proportions and to identify full siblings. Based on that, different models were applied to estimate the phenotypic difference and difference in mean genotypic values between Inuit and European genetic ancestry. For comparison, we also included Europeans in the form of 6514 Danish individuals from the population-based Inter99 cohort [22] to directly calculate the phenotypic difference between Inuit and Europeans. Overview of the phenotypes and phenotypic values is available in Table S1.

### Anthropometric and biochemical measurements

Anthropometric and biochemical data collection in the Greenlandic cohorts (B99, IHIT, B2018) and the Danish Inter99 cohort have been described previously [19–21, 23, 24]. In brief, body weight, height and waist and hip circumference were measured, and body mass index (BMI) and waist-to-hip ratio calculated in both the Greenlandic and the Danish cohorts, and in the Greenlanders also visceral and subcutaneous adipose tissue were measured and lean body mass and fat percentage calculated. In both the Greenlandic and the Danish cohorts fasting levels of HbA1c, serum total cholesterol, high-density lipoprotein (HDL) cholesterol, triglyceride, apolipoprotein A1 (ApoA1) and apolipoprotein B (ApoB) concentrations were measured, and levels of remnant cholesterol and LDL cholesterol were calculated. In the Greenlandic cohorts, participants not treated for diabetes with years of age >18 (IHIT) or ≥35 (B99, B2018), and in the Danish cohort, all participants without known diabetes, underwent an oral glucose tolerance test. To further eliminate the possible effect of treatment for diabetes, individuals with known diabetes were excluded from all analyses of measures of glucose homeostasis. Plasma glucose, serum insulin and serum C-peptide levels were measured at fasting and 2-h after ingestion of the glucose load. Information on birth weight and length were retrieved from midwife records for the Danish cohort, and from medical records at hospitals, including birth records, midwife records and outpatient records as well as information from the central birth register of Greenland for the Greenlandic cohorts.

### Whole genome sequencing

A subset of 448 Greenlandic individuals from IHIT and B99, selected based on sampling location independently of phenotype and disease status, underwent whole genome sequencing (WGS; Illumina) with an average sequencing depth of ∼35×. Reads were cleared for adapters using biobambam tools [25] and mapped with BWA-MEM [26] to GRCh38 (bwa mem -t 24 -p -Y -K 100000000). After mapping, duplicated reads were marked. Genotype calling was done using GATK haplotype caller and variant quality score recalibration (VQSR) tools based on the GATK resource bundle [27]. Only variants in the T98 tranche and above were used. The sites were parsed through Plink (v1.90b6) [28], keeping the two most common alleles of multiallelic sites.

### Genotyping and imputation

The Greenlandic samples that were not whole genome sequenced were genotyped using Multi-Ethnic Global Array (MEGA chip, Illumina), which was described previously in [7]. After quality control, it comprised about 1.6 million variants. To impute the missing data, we first inferred the relatedness among the WGS individuals using ancestry fractions and inferred ancestry-specific allele frequencies from Admixture [5, 28] with the assumed number of ancestral populations equal to two as input to NGSremix [29], which outputs pairwise relatedness coefficients. We then phased the WGS data using Shapeit2 [30] with trio information inferred from the relatedness results. Next, we used IMPUTE2 [30, 31] to pre-phase the participants with genotyping array data and then imputed them with merged reference of WGS and 1KGP (specific pops are CDX|CEU|CHB|CHS|GBR|TSI|IBS) [32]. The workflow is available at GitHub [33]. Sites in the imputed data with MAF below 0.05, missing call frequencies greater than 0.05 and high LD (*r*^2^ > 0.8) were filtered out, resulting in a dataset of 856,924 sites. We used this dataset to estimate ancestry proportions and pairwise relatedness.

### Genetic ancestry inference

We estimated ancestry proportions for the Greenlandic sample using ADMIXTURE with the assumed number of ancestral populations equal to two, which is an approach we have previously shown leads to accurate estimates of Inuit and European ancestry proportions [6, 7]. Which of the two ancestral populations that corresponds to Inuit was identified by comparing our results to those from [6], in which a panel of 50 Danes were included to allow for an unambiguous genetic ancestry component assignment.

### Relatedness estimation and sibling identification

With the ancestry proportions and genotype data as input, we estimated the relatedness between samples using the RelateAdmix method from [34] as implemented in NGSremix [29]. This method allows estimation of pairwise relatedness between admixed individuals as the fractions of loci sharing 0, 1, 2 alleles identical by descent (IBD; k0, k1, k2) for all pairs of individuals in a manner that takes admixture into account. Since the expected IBD sharing for full siblings as (k0, k1, k2) is (0.25, 0.5, 0.25), we visualised the distribution of k2 along k1 and chose the cluster with k1 between 0.25 and 0.75, and k2 between 0.15 and 0.5 as full siblings. Some pairs near the boundary, which had k1 between 0.4 and 0.6, and k2 above 0.125, were included because they are both full siblings to other individual(s) within the former cluster.

To estimate the genetic ancestry of the parents of the siblings, we also used NGSremix, which has an implementation of the method apoh [35] that provides such estimates.

### Estimation of the difference in mean phenotype values and in mean genotypic values between Inuit and Europeans

To quantify the phenotype differences between Inuit and Europeans, we first regressed the phenotype values with ancestry proportions of all the individuals from the admixed full-sibling pairs using the following regression model:

model 1a $${Y}_i=\alpha +{\beta}_Y{Q}_i+{\beta}_A Ag{e}_i+{\beta}_S Se{x}_i+{\epsilon}_i,$$

where *Y*_*i*_ and *Q*_*i*_ denote the phenotype value and the Inuit ancestry proportion, respectively, for individual *i*. *α* is the intercept. Recruitment age *Age*_*i*_ and sex *Sex*_*i*_ for individual *i* are included as covariates and their effect size are denoted as *β*_*A*_ and *β*_*S*_,respectively. The residual is represented as *ϵ*_*i*_ with *ϵ*_*i*_ ~ N(0,${\sigma}_{\epsilon}^2$) and importantly, *β*_*Y*_ is an estimate of the difference in the mean phenotype value (∆Y) between Inuit and Europeans. For clarity, we denote the estimated *β*_*Y*_ as $\hat{\varDelta {Y}_{SibQ}}$ in the “Results” section.

Similarly, we estimated the difference in mean genotypic value between Inuit and Europeans using the following regression model:

model 1b $$\partial {y}_j=\alpha +{\beta}_G\partial {Q}_j+{\beta}_{\partial age}\partial Ag{e}_j+{\beta}_{age1} Ag{e}_{j1}+{\beta}_{\partial sex}\partial Se{x}_j+{\beta}_{sex1} Se{x}_{j1}+{\epsilon}_j,$$

where *∂y*_*j*_ is the phenotype value difference between the two siblings in admixed full-sibling pair *j* and *∂Q*_*j*_ is the difference of Inuit ancestry proportion between the two siblings in admixed full-sibling pair *j*. The difference in age (*∂*Age_j_﻿), and the age of the first sibling (Age_j1_) are included as covariates. Similarly, their sexes are also included with *∂Sex*_*j*_ denoting the difference of sexes between them and *Sex*_*j*1_ denoting the sex of the first sibling. *α* is the intercept and the residual is *ϵ*_*j*_ ~ N(0,${\sigma}_{\epsilon}^2$). Importantly, here the estimate for *β*_*G*_ is an estimate of the difference in mean genotypic value (∆G) between Inuit and Europeans. For clarity, we denoted the estimate of *β*_*G*_ as $\hat{\varDelta {G}_{Siblings}}$ in the “Results” section.

The basic models above (model 1a and 1b) do not take into account that some of the sibling pairs are from the same family. Therefore, we also explored a model similar to the one recently used to evaluate within family polygenic score prediction [8]. Specifically, we explored the following regression model which adjusts for the family effect and overcome the correlation of multiple sibling pairs within a family:

model 2 $${y}_{ij}=\alpha +{\beta}_W\left({Q}_{ij}-\overline{Q_j}\right)+{\beta}_B\overline{Q_j}+{\beta}_{age} Ag{e}_{ij}+{\beta}_{sex} Se{x}_{ij}+{\gamma}_j+{\epsilon}_{ij},$$

where *y*_*ij*_ and *Q*_*ij*_ denote the phenotype value and Inuit ancestry proportion, respectively, for the admixed full sibling *i* in family *j*, and $\overline{Q_j}$ is the mean Inuit ancestry proportion in family *j*. *α* is the intercept. Age and sex are included as covariates and their effect size are denoted as *β*_*age*_ and *β*_*sex*_, respectively. *γ*_*j*_ denotes a family random effect with *γ*_*j*_ ~ N(0,${\sigma}_{\gamma}^2$). The residual is represented as *ϵ*_*ij*_ with *ϵ*_*ij*_ ~ N(0,${\sigma}_{\epsilon}^2$). Notably, in this model *β*_*B*_ is the difference in mean phenotypes for Inuit and Europeans (∆Y) and *β*_*W*_ is the difference in mean genotypic value for Inuit and Europeans (∆G). For clarity, we denoted *β*_*B*_ as $\hat{\varDelta {Y}_{FamilyQ}}$ and *β*_*W*_ as $\hat{\varDelta {G}_{Families}}$ in the “Results” section.

### Simulation-based sensitivity analysis

First, we investigated the direction and extent of the potential bias induced by ancestry-by-environment interactions on the estimates of the difference in mean phenotypic values (∆Y) and mean genotypic values (∆G). We first simulated a scenario, where the environment is correlated with the family’s average ancestry proportion and each sibling’s phenotype value is as ${y}_{ij}\sim N\left({Q}_{ij}\Delta G+{E}_{anc}\overline{Q_j},{\sigma}^2\right)$, where *E*_*anc*_ is the ancestry-by-environment interaction term. Because we do not expect siblings or their surroundings to know which of them have the higher or lower proportion of a certain ancestry, then we expect an ancestry-by-environment interaction to act on a family level and not on the individual level. However, if there is effect on the individual independently of the family, then we expect it to have an impact on our estimates. Therefore, we also simulated ancestry-by-environment interaction on the individual sibling’s admixture proportion level such that *y*_*ij*_~*N*(*Q*_*ij*_∆*G* + *E*_*anc*_*Q*_*ij*_, *σ*^2^). We explored varying values for *E*_*anc*_ with ∆G equals to 1 and *σ* equals to 1. We estimated the ∆Y using model 1a and estimated the ∆G using model 1b and compared them to the true difference in mean genotypic values. We used estimated *Q*_*ij*_ from our real data. We performed 10,000 simulations for each value of *E*_*anc*_.

To further explore possible bias, we simulated so-called participation bias where individuals with a higher phenotype value are less likely to participate in the study. This was done by choosing a threshold to define what a high phenotype value is. If an individual has a phenotype value above this threshold, there was a 20% chance that the individual would not participate in the study. If one individual does not participate, then the sibling pair containing this individual will be removed from estimation of ∆G. For families with more than 1 sibling pair, the remaining siblings who all participate will be kept. The phenotype values for all siblings were simulated as *y*_*ij*_~*N*(*Q*_*ij*_∆*G*, *σ*^2^). To keep the number of individuals and sibling pairs the same regardless of the degree of participation bias, a new phenotype was simulated for non-participating siblings based on their genetic ancestry until it was below the threshold phenotype value. We simulated different thresholds for having a high phenotype based on the quantiles of the simulated phenotypes. Similarly, we also explored another kind of participation bias where participation in the study depends on the phenotype and ancestry of the mother. The mothers’ ancestry proportion was estimated by NGSremix and was used to simulate the phenotype values of the mothers.

Finally, we performed simulations to investigate to what extent errors in the estimation of Q might affect our estimation of ∆G. The phenotypes were simulated as *y*_*ij*_~*N*(*Q*_*ij*_∆*G*, *σ*^2^), but errors were added to *Q*_*ij*_ when estimating ∆G. We performed 1 million simulations where the estimation error of *Q* was as ${\epsilon}_Q\sim N\left(0,{\sigma}_{Qerror}^2\ \right)$ and ${\sigma}_{Qerror}^2$ varied between 0 and 5%. We quantify the estimation error of *∂Q* in our data to be 0.24% based on F1s (see how the error of *∂Q* was estimated below), so our simulation range also includes values of estimation errors many folds larger than our estimated error. From there we estimated ∆*G* and reported the deviation from the true ∆*G* along with the absolute mean error in the difference of Inuit genetic ancestry. We tried 100 values randomly between 0 and 0.05 for ${\sigma}_{Qerror}^2$ and estimated ∆*G* using model 1b.

### Estimation of average error in estimates of *∂Q*

To estimate the average error in our estimation of the difference in ancestry proportions within each pair of full siblings, we took advantage of the fact that the dataset contained a number of F1 full-sibling pairs. For these pairs, each sibling must per definition have exactly 50% genetic ancestry from Inuit and exactly 50% genetic ancestry from Europe and true difference in their ancestry proportions must therefore be 0. This means that any estimated difference in genetic ancestry is due to error alone and we therefore estimated the average error in the estimated difference in ancestry proportions as the average absolute difference in genetic ancestry within all F1 sibling pairs.

## Results

### Identification of admixed full siblings

**Fig. 2:**
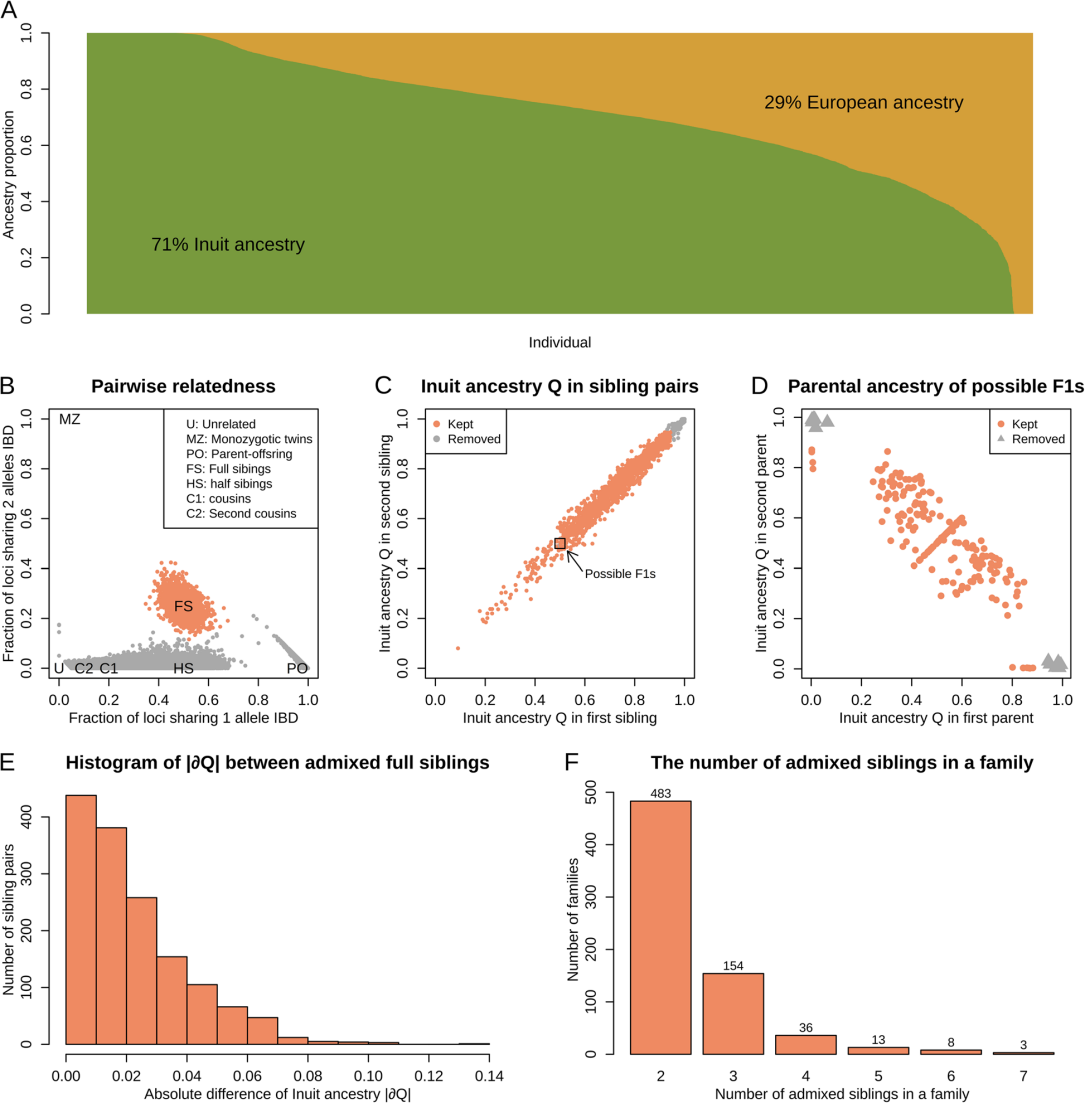
Identification of admixed full siblings. **A** Barplot illustrating the ancestry proportions inferred for all the Greenlandic individuals by ADMIXTURE assuming two ancestral populations. European genetic ancestry is shown in yellow. **B** Pairwise relatedness estimated by NGSremix. Full siblings (FS) are shown in orange. **C** Inuit ancestry proportions between full siblings. Based on this we removed all pairs, where both siblings had more than 95% of the same genetic ancestry (shown in grey). **D** Inuit ancestry proportions of the parents of potential F1 siblings, which are shown in the square in the centre in Fig. 2C. Siblings with parents that were unadmixed and from different populations (shown in grey) were removed since their offspring will have the exact same admixture proportion. **E** Histogram of absolute difference in Inuit ancestry proportion between admixed full siblings. **F** The number of admixed full siblings in a family

To identify admixed siblings, we first imputed genome-wide data from 5996 Greenlanders and based on 856,924 LD-pruned common variants (MAF >5%), we estimated their ancestry proportions using ADMIXTURE. On average, we observed 29% European genetic ancestry, but there was substantial variation between individuals (Fig. 2A).

Next, we identified all pairs of full siblings using NGSremix (Fig. 2B), which estimates the relatedness coefficients k1 and k2, while taking admixture into account. Full siblings clustered around (k1=0.5, k2=0.25) and in total we found 1869 full-sibling pairs. From these, we excluded all pairs of siblings where both parents were unadmixed, since there should not be any difference in the ancestry proportions between these siblings. To do so, we first excluded all pairs of full siblings where both siblings had more than 95% of the same genetic ancestry (Fig. 2C), representing those siblings whose parents were unadmixed with the same genetic ancestry. Second, we performed an additional analysis of the pairs of siblings where both had around 50% of each genetic ancestry (Fig. 2C), which might suggest unadmixed parents of different genetic ancestry. From these siblings, we estimated the admixture proportions of the parents, and we removed the sibling pairs, where both parents had more than 95% of a certain genetic ancestry (Fig. 2D), representing those siblings whose parents were unadmixed with different genetic ancestries (F1 siblings). This led to a final set of 1474 admixed full-sibling pairs for downstream analyses. Most of these full siblings differed by up to 5% in ancestry proportions and the difference ranged from 0.0043 to 13.1% (Fig. 2E), hence, we report the estimated difference in genotypic value per percentage point difference in genetic ancestry to avoid extrapolating effects outside the range of genetic ancestry differences observed in our data (Fig. S1). The full-sibling pairs were from 697 families and most of them were the only pair in their family (Fig. 2F).

### Metabolic phenotypes differ between Inuit and European genetic ancestry

We investigated differences in mean phenotype values between Inuit and European genetic ancestry across 26 metabolic phenotypes, based on the 1474 admixed Greenlandic full-sibling pairs.

Using the basic regression model outlined in Fig. 1C and D (model 1a in “Methods”), 13 out of the 26 phenotypes were estimated to differ significantly between Inuit and European genetic ancestry, and 12 of them remained significant after FDR correction for multiple testing (Table 1 and Table S1). Effect estimates for phenotype differences across measures of body composition indicated that individuals with high Inuit genetic ancestry were in general smaller than individuals with high European genetic ancestry, and the mean phenotype values were estimated to be significantly lower in Inuit genetic ancestry than European genetic ancestry for height (effect size per percentage of Inuit genetic ancestry (SE), −0.14 (0.01) cm/%), body weight (−0.16 (0.02) kg/%), hip circumference (−0.09 (0.02) cm/%), fat percentage (−0.09 (0.02) %/%), lean body mass (−0.07 (0.02) kg/%) and subcutaneous adipose tissue (−0.02 (0.004) cm/%), but higher waist-to-hip ratio (0.0006 (0.0001)/%). With respect to measures of glucose homeostasis, the mean phenotype values in Inuit genetic ancestry were estimated to be significantly lower for levels of 2-h plasma glucose (−0.008 (0.006) mmol/l/%), fasting serum insulin (−0.35 (0.08) pmol/l/%) and 2-h serum insulin (−1.4 (0.6) pmol/l/%), but higher levels of HbA1c (0.003 (0.001) %/%). Of note, the difference in 2-h glucose level was not significant after FDR adjustment for multiple testing. We also assessed lipid measures, here we found that the mean phenotypic levels of HDL cholesterol (0.003 (0.0008) mmol/l/%) and ApoA1 (0.004 (0.001) mmol/l/%) were estimated to be significantly higher in Inuit genetic ancestry. Using a more complex model, which takes into account that some of the sibling pairs are from the same family (model 2 in “Methods”), we got similar effect estimates, but the association with 2-h plasma glucose was no longer significant (Table 1 and Table S1).

**Table 1:** Estimated difference in mean phenotype between Inuit and European genetic ancestry

Phenotype	Estimated from admixed siblings in Greenland					
	***N***	Mean	$\hat{\varDelta {Y}_{SibQ}}$ (SE)/% model 1a	*P* value_SibQ_ (SD)	$\hat{\varDelta {Y}_{FamilyQ}}$(SE)/% model 2	*P* value_FamilyQ_ (SD)
Body mass index (kg/m^2^)	1627	27.0	−0.013(0.009)	0.058	−0.013(0.01)	0.12
Height (cm)	1638	161.5	−0.14(0.01)	8.5×10^−45^	−0.15(0.012)	9.7×10^−29^
Body weight (kg)	1628	70.6	−0.16(0.02)	3.6×10^−11^	−0.16(0.029)	3.6×10^−8^
Waist circumference (cm)	1614	92.1	−0.017(0.02)	0.35	−0.015(0.027)	0.5
Hip circumference (cm)	1614	99.4	−0.091(0.02)	5.6×10^−9^	−0.09(0.019)	1.2×10^−6^
Waist to hip ratio	1613	0.92	6e−04(0.0001)	2.1×10^−7^	0.00063(0.00015)	3.0×10^−6^
Fat percentage	901	30.5	−0.086(0.02)	7.2×10^−6^	−0.086(0.023)	2.0×10^−4^
Lean body mass (kg)	576	48.2	−0.072(0.02)	2.6×10^−6^	−0.08(0.019)	2.6×10^−5^
Visceral adipose tissue (cm)	574	7.3	−0.0041(0.006)	0.54	−0.006(0.0072)	0.47
Subcutaneous adipose tissue (cm)	573	3.2	−0.016(0.004)	2.0×10^−5^	−0.017(0.0048)	2.0×10^−4^
Birth weight (g)	300	3424.7	−3.0(2.6)	0.25	−3.2(3.3)	0.33
Birth length (cm)	271	51.4	−0.018(0.01)	0.12	−0.02(0.013)	0.14
Fp glucose (mmol/l)	1178	5.78	0.00048(0.002)	0.52	0.00078(0.0027)	0.66
2h-p glucose (mmol/l)	1059	6.00	−0.008(0.006)	0.044	−0.0077(0.0063)	0.074
Fs C-peptide (pmol/l)	1018	559.9	−1.4(0.7)	0.093	−1.5(0.84)	0.099
2h-s C-peptide (pmol/l)	880	1883.2	4.0(2.6)	0.14	3.7(3.1)	0.26
Fs insulin (pmol/l)	1178	53.58	−0.35(0.08)	1.4×10^−5^	−0.37(0.092)	7.6×10^−5^
2h-s insulin (pmol/l)	1059	212.6	−1.4(0.6)	0.0025	−1.3(0.65)	0.01
HbA1c (%)	1366	5.89	0.0026(0.001)	4.8×10^−4^	0.0027(0.0012)	0.0022
Fs HDL cholesterol (mmol/l)	1650	1.64	0.0032(8e−04)	3.3×10^−5^	0.0032(0.00093)	3.2×10^−4^
Fs LDL cholesterol (mmol/l)	1435	3.64	−0.00075(0.002)	0.52	−9e−04(0.0025)	0.56
Fs Triglyceride (mmol/l)	1473	1.27	−0.0041(0.001)	0.055	−0.0043(0.0015)	0.085
Fs Total cholesterol (mmol/l)	1611	5.87	0.0039(0.002)	0.06	0.0038(0.0022)	0.098
Fs Remnant cholesterol (mmol/l)	1135	0.52	−0.0012(0.0006)	0.24	−0.0012(0.00067)	0.27
Fs Apolipoprotein A1 (mmol/l)	432	1.72	0.0043(0.001)	1.9×10^−5^	0.0043(0.0012)	2.2×10^−4^
Fs Apolipoprotein B (mmol/l)	432	0.91	0.0013(0.0009)	0.4	0.0013(0.0012)	0.48

Difference in mean phenotype value ∆*Y* between Inuit and European genetic ancestry based on the admixed Greenlandic siblings. Estimates are based on either the slope between individual’s admixture proportions and phenotype value ∆*Y*_*SibQ*_, or based on the differences between mean admixture proportion of each family and their mean phenotype ∆*Y*_*FamilyQ*_. Effect size and SE estimates are shown by per percentage of Inuit genetic ancestry. The numbers of individuals are the same for both analyses. *P* values were calculated based on quantile transformed trait values. F, fasting; p, plasma; s, serum

The above analyses were based only on admixed Greenlanders, for comparison, and as a check of robustness, we also investigated differences in mean phenotype values for 22 of the metabolic phenotypes between the 1706 admixed Greenlanders (the individuals in the 1474 admixed full-sibling pairs) and up to 6514 Danes of similar age from the independent population-based Inter99 cohort. For most of the phenotypes showing significant differences in the analyses of admixed Greenlandic full-sibling pairs, we observed the same direction for effect estimates, however, for some phenotypes, we observed a different magnitude of effect, including hip circumference, 2-h serum insulin, HbA1c and ApoA1, and for fasting serum insulin the effect estimate was in the opposite direction (Table 1 and Table S1).

### Differences in mean genotypic value between Inuit and European genetic ancestry

**Fig. 3:**
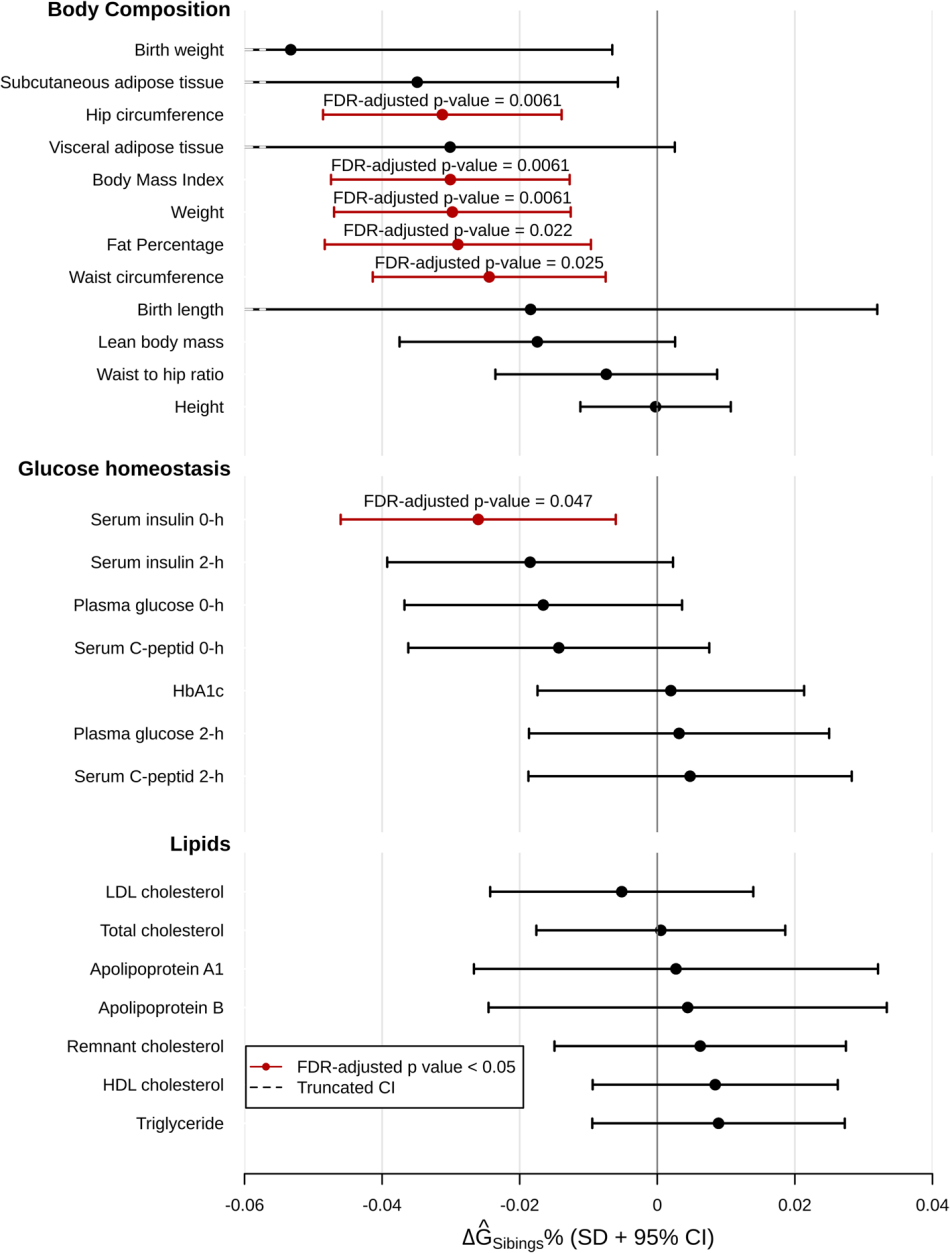
Estimation of difference in mean genotypic value between Inuit and Europeans. Estimates of difference in mean genotypic value between Inuit and European genetic ancestry, $\hat{\varDelta {G}_{Siblings}}$, obtained from admixed full-sibling pairs from Greenland with the basic regression model shown in Fig. 1B, C (using model 1b). Effects were estimated for quantile transformed phenotypes using a model with age and sex as covariates. Error bars are the 95% confidence intervals. Red colour means FDR adjusted *p* value < 0.05. Symmetry of the bars was broken around the borders due to the chopping of the *x*-axis for better presentation, which is indicated by the dashed line at the end of the bars

We analysed the difference in mean genotypic value across the 26 metabolic phenotypes using the basic regression model outlined in Fig. 1A and B (model 1b in “Methods”). The assumed linear relationship was supported by the model’s residuals along fitted values (Fig. S2). For six of the 26 phenotypes, the estimated difference in mean genotypic value between Inuit and European genetic ancestry remained significant after FDR adjustment (Fig. 3 and Table S2). Most of these differences were observed for phenotypes related to body composition, where Inuit genetic ancestry was associated with lower genotypic value for body weight (effect size per percentage of Inuit genetic ancestry (se), −0.51 (0.16) kg/%), BMI (−0.20 (0.06) kg/m^2^/%), waist circumference (−0.42 (0.16) cm/%), hip circumference (−0.38 (0.11) cm/%) and fat percentage (−0.38 (0.13) %/%). We did not observe a significant difference in mean genotypic value for the remaining phenotypes related to body composition, including height, for which we observed a relatively large difference in mean phenotype values of 0.14(0.01) cm/% (Table 1). We tested if this discrepancy could be explained by the recent changes in environmental exposures, likely primarily diet, by assessing height according to birth year. We observed that individuals born in 1990 were an estimated 12.4 cm taller than those born in 1930 (Fig. S3). Besides phenotypes related to body composition, we only observed a significant difference in mean genotypic value for fasting insulin (−1.07 (0.51) pmol/l/%) between Inuit and European genetic ancestry (Fig. 3 and Table S2).

We also estimated the differences in mean genotypic value with a more complex model, which takes into account that some of the sibling pairs are from the same family (model 2 in methods). With this model, we obtained similar estimates of mean genotypic value for most phenotypes (Fig. S4). When further FDR adjusting these analyses for multiple testing, the difference in waist circumference and fasting insulin was no longer significant (Table S2).

**Fig. 4:**
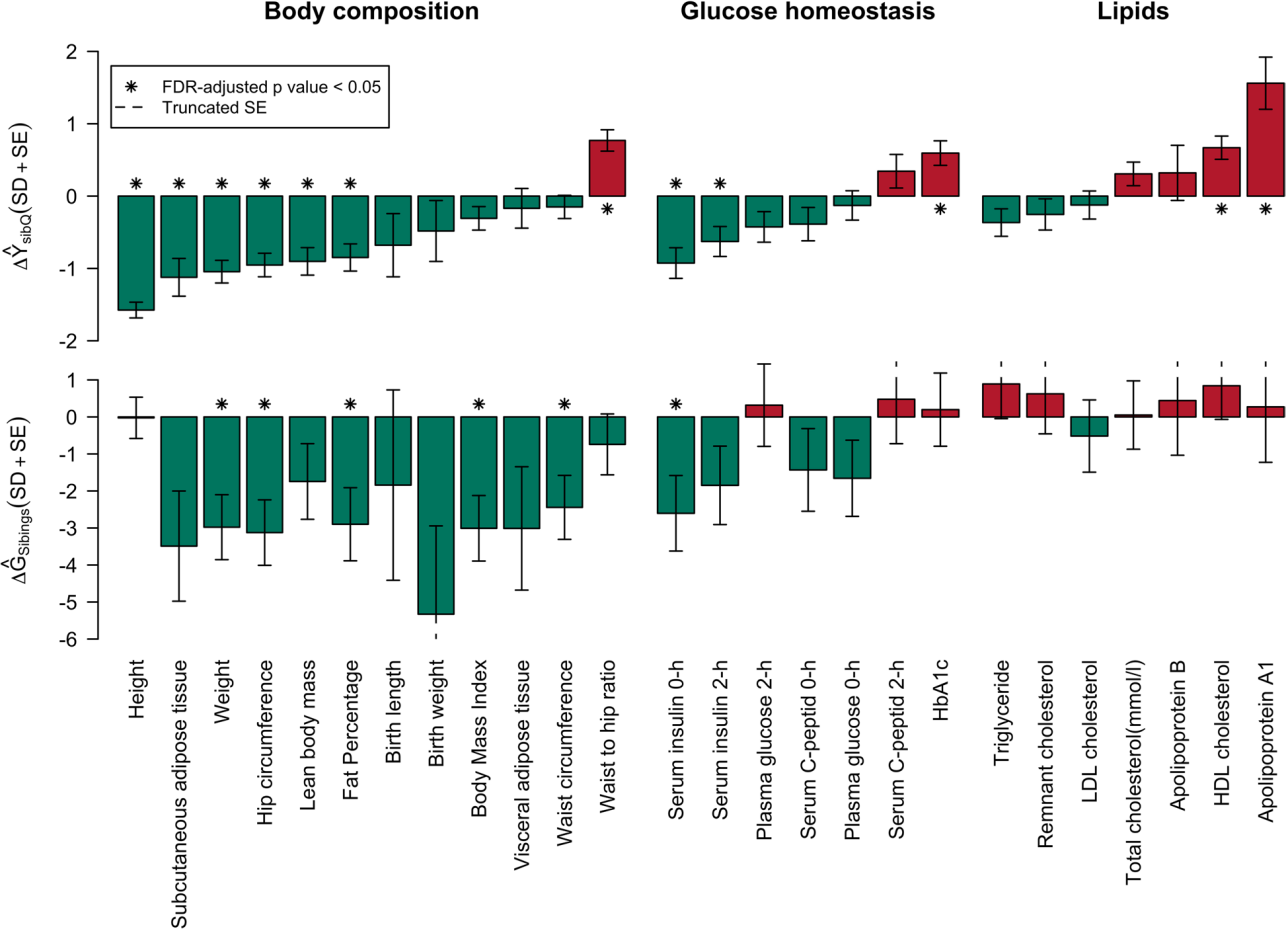
Directions of $\hat{\varDelta {Y}_{SibQ}}$ and $\hat{\varDelta {G}_{Siblings}}$ with quantile transformation. For each phenotype there is a bar that illustrates the point estimates for $\hat{\varDelta {Y}_{SibQ}}$ (top) and $\hat{\varDelta {G}_{Siblings}}$ (bottom) measured in standard deviations for easy comparisons across phenotypes. The whiskers indicate standard errors, and the colour of the bar indicates whether the point estimate is higher in Inuit genetic ancestry (red) or lower (green). Stars were added above/below the bar to indicate those whose FDR adjusted *p* values < 0.05. Symmetry of the bars was broken around the borders due to the chopping of the *y*-axis for better presentation, which is indicated by the dashed line at the end of the bars

Across models, only the differences in mean genotypic value for BMI, body weight, hip circumference and fat percentage passed FDR correction for multiple testing. However, importantly, all the significant differences in mean genotypic value showed the same direction as the difference in mean phenotypic values between Inuit and European genetic ancestry (Fig. 4).

### Sensitivity and robustness analysis

To investigate whether potential ancestry-by-environment interaction would lead to a bias in our estimates of the difference in mean genotypic value (∆G) when using our admixed sibling design, we simulated the ancestry-by-environment interaction effect using our real data’s estimated ancestry proportions. The simulations showed that the estimates were not confounded when the environment effect was correlated with the siblings’ average ancestry proportion within a family (Fig. S5). The simulations also showed that our design would lead to an overestimation of the difference for the mean genotypic values, if there was individually ancestry correlated environmental effects independently of its family’s genetic ancestry. However, this bias would be very limited relative to the variance in the estimates (Fig. S6) and much lower than for phenotypic difference (∆Y), where ancestry-by-environmental interaction had a large effect.

Further, we used simulation to assess the potential effect of participation bias where individuals with a certain phenotype were less likely to participate in the study. These simulations showed an underestimation of the absolute value of mean phenotypic values as expected, but little bias in the estimation of the difference in mean genotypic values (Fig. S7, S8) because it only uses information based on the difference within the admixed sibling pair.

Finally, since the ancestry proportions used for estimating the difference in mean genotypic values were estimated from genetic data, it will not be the true values. Therefore, we performed simulations to investigate how errors in genetic ancestry estimation can affect the estimates of the difference in mean genotypic values between the two ancestral populations. The simulations revealed that inaccurate estimation of Inuit ancestry proportion will lead to underestimation of $\hat{\varDelta G\ }$(Fig. S9). The size of the bias depends on the absolute size of the error, the larger the error, the larger the bias. Based on the inferred F1s, we estimated that the average error of *∂Q* in our study was 0.24%. In our simulations, this size of error leads to a slight underestimation of $\hat{\varDelta G}$ of 0.4%.

## Discussion

In this study, we estimated differences in mean phenotype values and mean genotypic values for metabolic phenotypes between Inuit and European genetic ancestry using a novel study design based on admixed full-sibling pairs. As previously observed, Inuit and European genetic ancestry differed with respect to mean phenotype values of most metabolic phenotypes, with the mean phenotypic value generally being smaller for Inuit genetic ancestry than European genetic ancestry. In line with this, we showed that Inuit genetic ancestry was associated with significantly smaller mean genotypic values, primarily for body composition-related phenotypes and also fasting insulin levels. However, surprisingly for height, we observed a large difference in mean phenotype value between Inuit and European genetic ancestry, but no significant difference in mean genotypic value.

We showed that there is a significant difference in mean phenotype values between Inuit and European genetic ancestry for 13 out of the 26 metabolic phenotypes we analysed in the admixed Greenlanders. Particularly for measures of body composition, we observed lower mean phenotype values for Inuit genetic ancestry. These findings were supported by analyses comparing the phenotype values in the admixed Greenlandic siblings with Danes, and also by previous studies showing the same directions of effect estimations for fat percentage, height, body weight, hip circumference, triglyceride levels and 2-h serum insulin levels [24, 36, 37]. However, we observed larger magnitudes of effect in our study of admixed siblings, likely because in previous studies participants recruited as non-admixed ethnicities, may contain admixed individuals, which would underestimate the difference between ethnicities. Of note, for fasting insulin we observed opposite directions of effect when estimating the mean phenotype difference between Inuit and European genetic ancestry based on the admixed Greenlandic siblings and by comparing the admixed Greenlandic siblings with the Danes from the independent Inter99 cohort. This discrepancy could be caused by environmental differences between Greenland and Denmark.

For mean genotypic values, we primarily observed differences between Inuit and European genetic ancestry for phenotypes related to body composition, namely body weight, fat percentage, hip circumference, waist circumference and BMI. For these phenotypes, our results indicated that differences in mean genotypic values at least partly explained the difference in phenotype values, as Inuit genetic ancestry both had lower phenotype values and lower mean genotypic values. For body weight, this is consistent with previous observations of alleles having significantly negative effect sizes in Inuit but little or no effect in Europeans [13]. We did not necessarily expect the same direction of effect for estimates of difference in mean phenotype and mean genotypic values across traits, as effects in opposite directions can easily occur unless the two populations that are being compared have the exact same environment [2]. Notably, it has also been shown that differences in mean genotypic values between two populations can occur both under neutrality and under selection [2]. Hence, importantly, differences in mean genotypic values between populations or ancestries do not imply that one population or genetic ancestry is superior to the other in terms of fitness in any given environment.

With respect to height, we observed a very significant difference in the mean phenotype values between Inuit and European genetic ancestry, but no difference in mean genotypic values. This is consistent with previous observations of alleles having similar effect sizes for height in both Inuit and Europeans [13]. This discrepancy between difference in mean phenotype value and mean genotypic value observed for height is likely driven by recent changes in environmental exposures, including the general Greenlandic diet and a reduction in infectious-disease burden, which pushes the mean height in the population towards the genetic potential for the population. This was supported by our observation of significant correlation between birth year and height, where Greenlanders born in 1990 were substantially taller than those born in 1930, and by a previous study showing that Greenland is among the countries with the largest increase in human height from 1896 to 1996 [1]. Also, it has previously been shown that dietary quality in Greenland is significantly associated with a higher socio-economic position and higher degree of urbanisation [38], and that higher socio-economic position is significantly associated with greater height [39]. Similarly, we observed differences in mean phenotype values for lipid phenotypes, but no differences in mean genotypic values, suggesting that the differences in these mean phenotypic values are caused by differences in environmental exposures alone. This is supported by a previous study showing that cholesterol and triglyceride varied according to westernisation, diet, alcohol consumption and smoking [37].

Our simulations showed that our admixed sibling design leads to a bias in the estimates of the difference in mean genotypic value due to the fact that the true ancestry proportions are not known, but were inferred. However, this bias was small and importantly, our simulations also showed that our estimation of difference in mean genotypic value between ancestries remains quite robust in regard to differences in environmental exposure between siblings. This robustness towards environmental confounding is one of the great advantages of our method compared to methods testing the correlation between a phenotype and the degree of genetic ancestry [40, 41]. We note that a sibling design, similar to ours, has been explored where PGS are used instead of genetic ancestry [8, 42]. And importantly, Abdellaoui et al. [42] recently showed that the effect of PGS within a family can be confounded by regional environmental differences. Although this confounding is not large, this result suggests that sibling designs might not be entirely robust. However, we emphasise that admixture proportions are generally not easily confounded unlike PGS, which are easily confounded by genetic ancestry. Hence, all in all the admixed sibling design proposed in this study can be confounded, but the effects of the confounding seem to be relatively small, suggesting that the design is quite robust.

Another major strength of the study is the inclusion of a large proportion of the Greenlandic population with precise measures of phenotypes. However, although we have genetic data for more than 10% of the adult present-day Greenlandic population in our dataset, the sample size varied across the investigated phenotypes, and we only found six phenotypes, BMI, body weight, waist circumference, hip circumference, fat percentage and fasting serum insulin, with a significant difference in mean genotypic value between ancestries after FDR correction for multiple testing. And notably, some of the confidence intervals for the effect size estimates were wide, suggesting that an even larger sample size would be needed to fully assess exactly which phenotypes have significant differences in mean genotypic value. It is also worth noting that our estimates of the difference in mean genotypic values (∆G) for body weight was unrealistically high between Inuit and European genetic ancestry, compared to the difference in mean body weight (∆Y) between these two ancestries. The reason is most likely due to extrapolating outside the data range for difference of ancestry proportion between the full siblings that were analysed. Another possible limitation of our study is the possible confounding by treatment, as we only accounted for the use of glucose-lowering medication.

To more fully explore the potential of the presented novel study design, it would be very useful to increase the sample size in Greenland even further. Also, it would be interesting to apply it to other admixed populations. However, we emphasise that our current approach can only be applied to admixed populations that are the result of a simple two-way admixture between populations that are so genetically distinct that admixture proportions can be accurately estimated. Thus, we acknowledge that this method cannot be applied to all admixed populations unless better data or methods are available for genetic ancestry estimation. We also acknowledge that there is a risk that the method could be misused with the purpose of showing that genetics contribute to differences that can be argued to make some populations superior to other populations, e.g. in terms of educational attainment. We of course highly discourage such misuse and hope that the method will instead, like other family-based studies, be used to disprove such hypotheses because they are more robust to environmental differences [11, 43, 44].

## Conclusion

In conclusion, this study is the first to show that differences in mean genotypic values do explain part of the differences in the mean value of metabolic phenotypes observed between any two human populations. Comparing genetic Inuit and European genetic ancestry, we primarily observed differences in mean genotypic values for phenotypes related to body composition, but also fasting insulin. We leveraged the unique genetic properties of the highly admixed Greenlandic population, and by focusing on this underrepresented population, we were able to estimate differences in mean genotypic value based on admixed full-sibling pairs, which markedly reduces the confounding by environmental exposures.

### Supplementary Information

**Additional file 1.** Table S1: Phenotypic difference between Inuit and Europeans. Effect sizes, standard errors and FDR adjusted p values of the estimated phenotypic difference from different models and cohorts. Table S2: Difference in mean genotypic values between Inuit and Europeans. Effect size, standard errors and FDR adjusted p values of the estimated difference in mean genotypic values from different models.

**Additional file 2.** Figure S1-S9.PDF file with all supplementary figures S1-S9 and corresponding legends.

## Data Availability

The datasets analysed during the current study are available in the European Genome-phenome Archive, under the accessions EGAD00010002057 [45].

## Abbreviations

ApoA1: Apolipoprotein A1
ApoB: Apolipoprotein B
BMI: Body mass index
HDL: High-density lipoprotein
IBD: Identical by descent
LDL: Low-density lipoprotein
PGS: Polygenic score
WGS: Whole genome sequencing
