# Supplementary material for: Analysis of admixed Greenlandic siblings shows that the mean genotypic values for metabolic phenotypes differ between Inuit and Europeans
Source: Genome Med. 2024 May 23;16:71. doi: 10.1186/s13073-024-01326-3 (PMC11112775; doi:10.1186/s13073-024-01326-3)
Supplement: Supplementary file 2 — Additional file 2. Figure S1-S9.PDF file with all supplementary figures S1-S9 and corresponding legends. [file 13073_2024_1326_MOESM2_ESM.pdf]

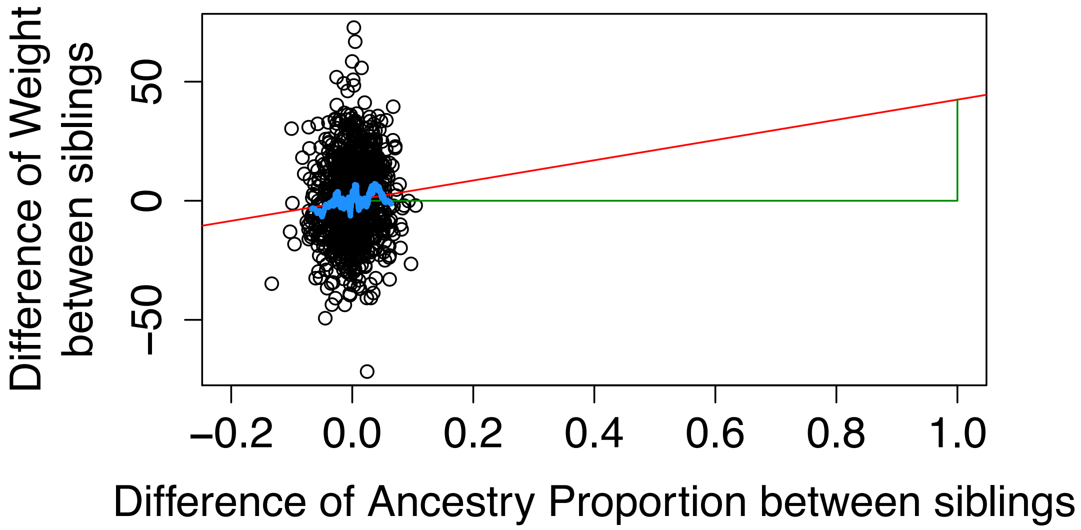

Figure S1. Illustration of the issue with extrapolating outside of the data range for weight. The difference of European ancestry proportions between siblings varies from -0.133 to 0.105. The blue lines are the sliding means of difference of weight along difference of ancestry proportion in a step of 50. The red line represents the linear regression based on the data. The horizontal green line is between 0 and 1. The vertical green line represents the overestimated difference in genotypic values between European and Inuit ancestries due to missing data between 0.105 and 1 for x axis.

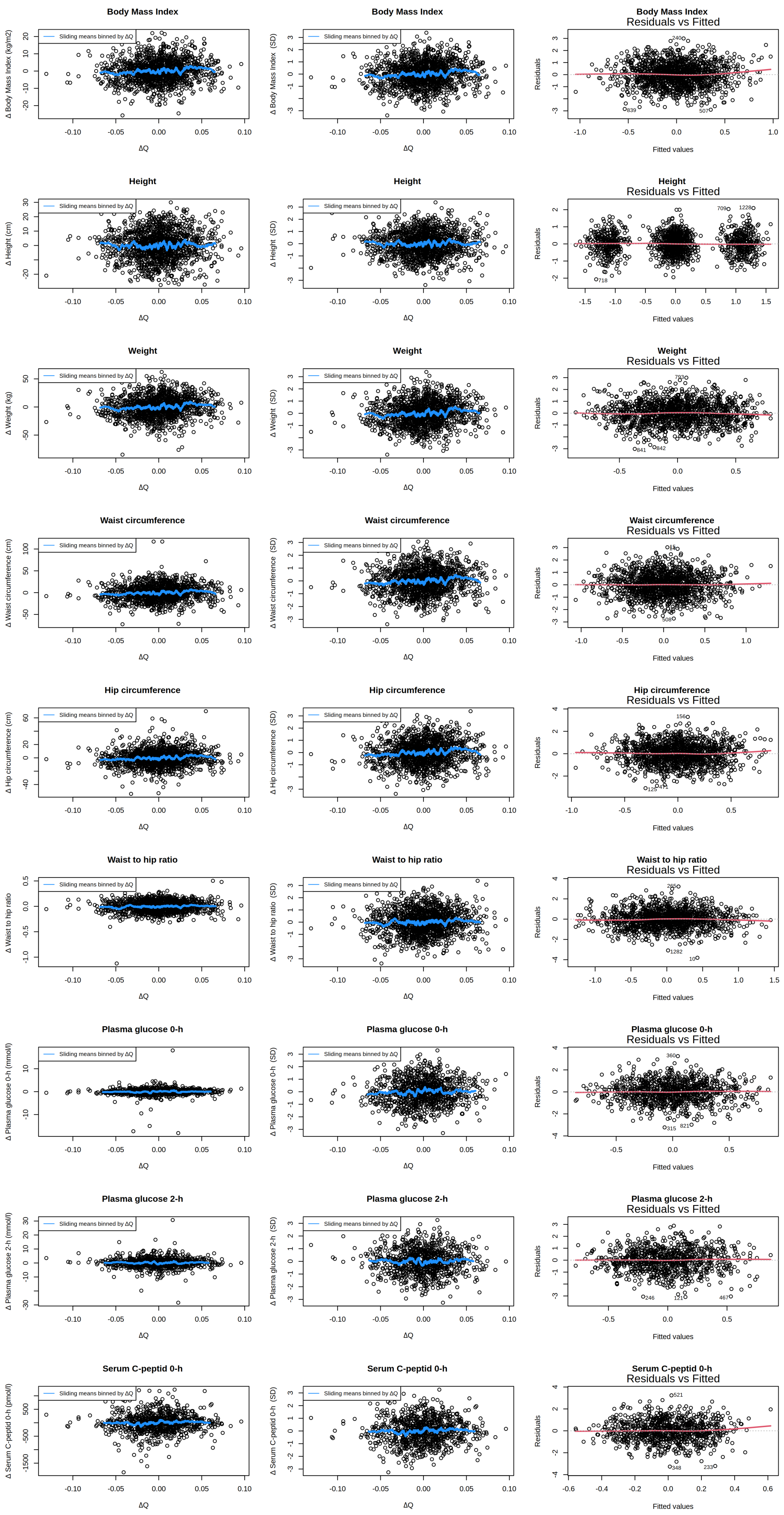
Body Mass Index
Sliding means binned by ΔQ
Δ Body Mass Index (kg/m2)
ΔQ
Body Mass Index
Δ Body Mass Index (SD)
Body Mass Index
Residuals vs Fitted
Residuals
Fitted values
Height
Δ Height (cm)
Height
Δ Height (SD)
Height
Residuals vs Fitted
Weight
Δ Weight (kg)
Weight
Δ Weight (SD)
Weight
Residuals vs Fitted
Waist circumference
Δ Waist circumference (cm)
Waist circumference
Δ Waist circumference (SD)
Waist circumference
Residuals vs Fitted
Hip circumference
Δ Hip circumference (cm)
Hip circumference
Δ Hip circumference (SD)
Hip circumference
Residuals vs Fitted
Waist to hip ratio
Δ Waist to hip ratio
Waist to hip ratio
Δ Waist to hip ratio (SD)
Waist to hip ratio
Residuals vs Fitted
Plasma glucose 0-h
Δ Plasma glucose 0-h (mmol/l)
Plasma glucose 0-h
Δ Plasma glucose 0-h (SD)
Plasma glucose 0-h
Residuals vs Fitted
Plasma glucose 2-h
Δ Plasma glucose 2-h (mmol/l)
Plasma glucose 2-h
Δ Plasma glucose 2-h (SD)
Plasma glucose 2-h
Residuals vs Fitted
Serum C-peptid 0-h
Δ Serum C-peptid 0-h (pmol/l)
Serum C-peptid 0-h
Δ Serum C-peptid 0-h (SD)
Serum C-peptid 0-h
Residuals vs Fitted

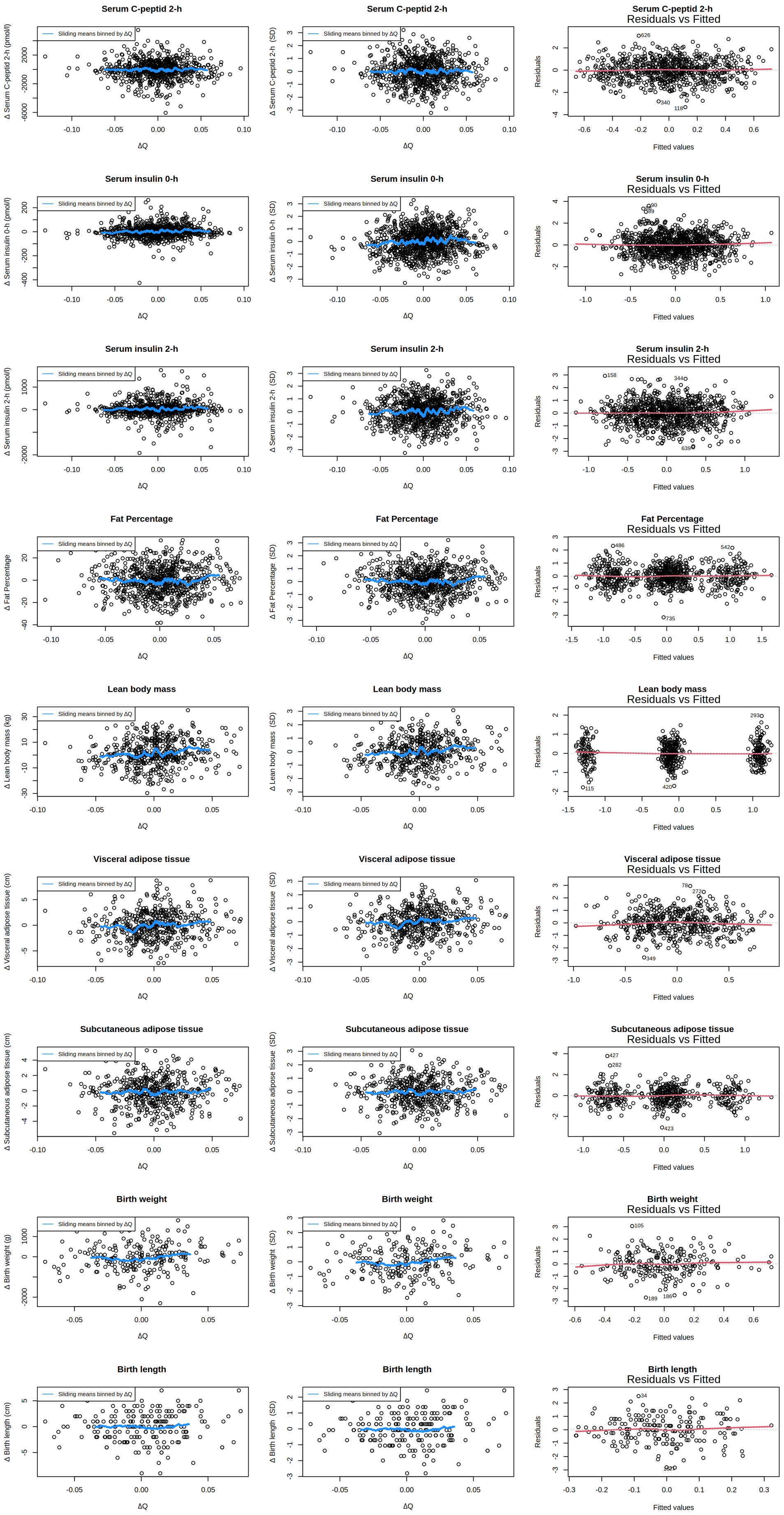
Serum C-peptid 2-h
Δ Serum C-peptid 2-h (pmol/l)
Δ Serum C-peptid 2-h (SD)
Serum C-peptid 2-h
Residuals vs Fitted
Serum insulin 0-h
Δ Serum insulin 0-h (pmol/l)
Δ Serum insulin 0-h (SD)
Serum insulin 0-h
Residuals vs Fitted
Serum insulin 2-h
Δ Serum insulin 2-h (pmol/l)
Δ Serum insulin 2-h (SD)
Serum insulin 2-h
Residuals vs Fitted
Fat Percentage
Δ Fat Percentage
Δ Fat Percentage (SD)
Fat Percentage
Residuals vs Fitted
Lean body mass
Δ Lean body mass (kg)
Δ Lean body mass (SD)
Lean body mass
Residuals vs Fitted
Visceral adipose tissue
Δ Visceral adipose tissue (cm)
Δ Visceral adipose tissue (SD)
Visceral adipose tissue
Residuals vs Fitted
Subcutaneous adipose tissue
Δ Subcutaneous adipose tissue (cm)
Δ Subcutaneous adipose tissue (SD)
Subcutaneous adipose tissue
Residuals vs Fitted
Birth weight
Δ Birth weight (g)
Δ Birth weight (SD)
Birth weight
Residuals vs Fitted
Birth length
Δ Birth length (cm)
Δ Birth length (SD)
Birth length
Residuals vs Fitted
Sliding means binned by ΔQ
ΔQ
Residuals
Fitted values

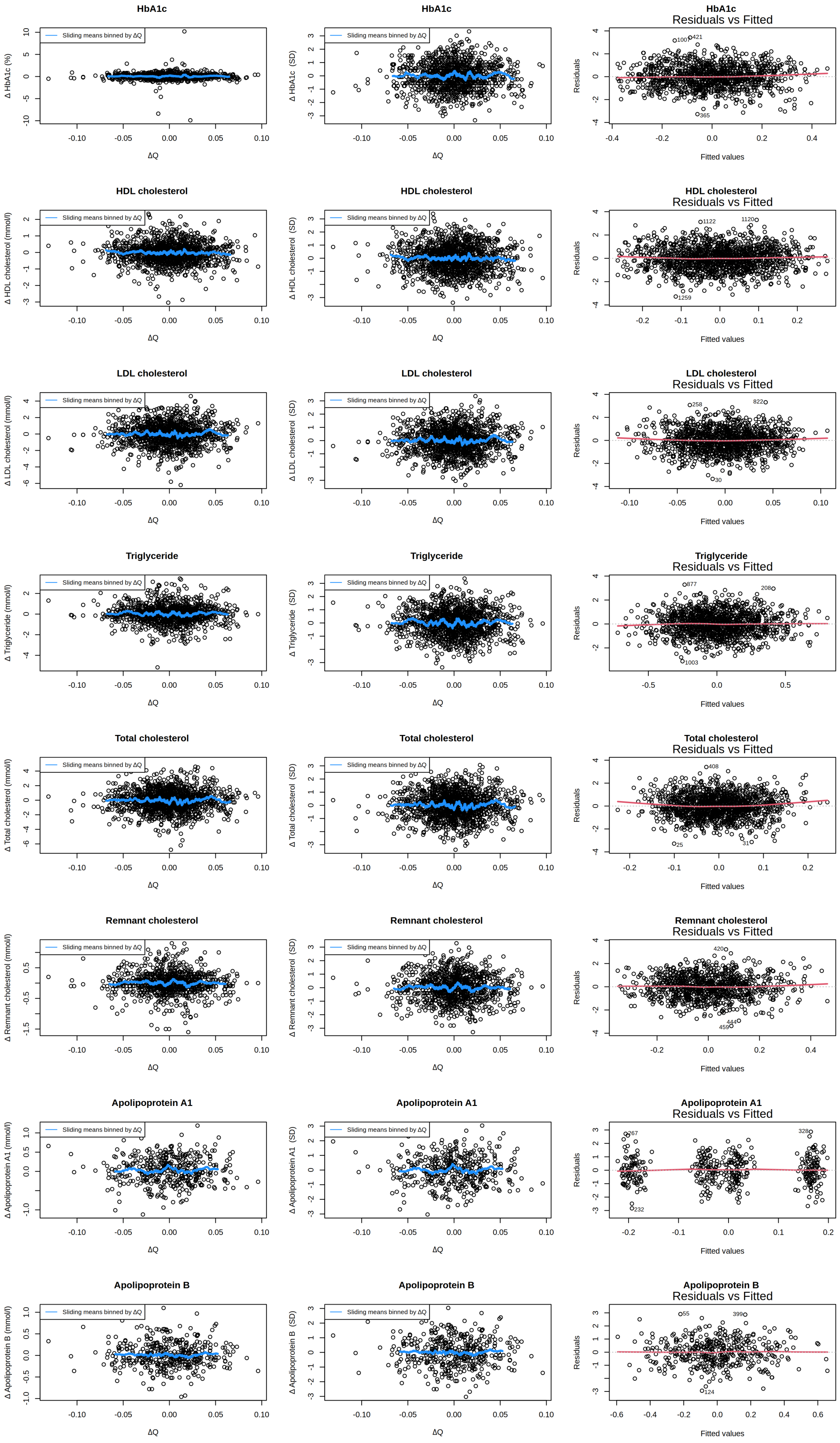
HbA1c
Δ HbA1c (%)
Δ HbA1c (SD)
HbA1c
Residuals vs Fitted
HDL cholesterol
Δ HDL cholesterol (mmol/l)
Δ HDL cholesterol (SD)
HDL cholesterol
Residuals vs Fitted
LDL cholesterol
Δ LDL cholesterol (mmol/l)
Δ LDL cholesterol (SD)
LDL cholesterol
Residuals vs Fitted
Triglyceride
Δ Triglyceride (mmol/l)
Δ Triglyceride (SD)
Triglyceride
Residuals vs Fitted
Total cholesterol
Δ Total cholesterol (mmol/l)
Δ Total cholesterol (SD)
Total cholesterol
Residuals vs Fitted
Remnant cholesterol
Δ Remnant cholesterol (mmol/l)
Δ Remnant cholesterol (SD)
Remnant cholesterol
Residuals vs Fitted
Apolipoprotein A1
Δ Apolipoprotein A1 (mmol/l)
Δ Apolipoprotein A1 (SD)
Apolipoprotein A1
Residuals vs Fitted
Apolipoprotein B
Δ Apolipoprotein B (mmol/l)
Δ Apolipoprotein B (SD)
Apolipoprotein B
Residuals vs Fitted
Sliding means binned by ΔQ
ΔQ
Residuals
Fitted values

Figure S2. Linear relationship between ∂Y and ∂Q. In each row are three plots for one trait. From left to right are (1) raw data distribution of ∂Y along ∂Q, (2) quantile-transformed ∂Y along ∂Q, (3) residuals along fitted values from model 1b using data in (2). The blue lines are sliding means in a step of 50 binned by the variable in x axis. The red lines are locally weighted scatterplot smoothing curves to help identify patterns in the residuals.

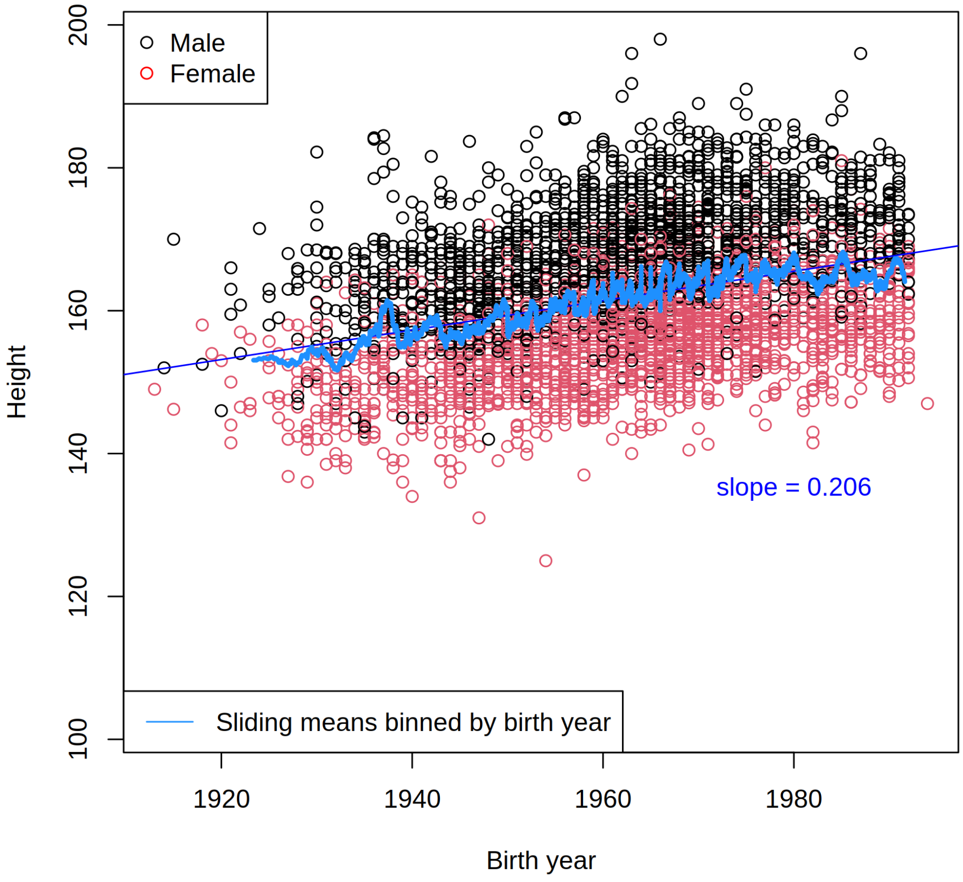

Figure S3. Distribution of height according to birth year. Birth year of participants were obtained by sampling year subtracting age of participants recorded when sampled, along with height. The blue lines are the sliding means of difference of height along birth year in a step of 50. We used 2010 as an approximate sampling year for IHIT, 1999 for B99, and 2018 for B2018. A linear regression between height and birth year for all individuals shows an increase of 0.206 cm per year. Individuals are coloured by sex.

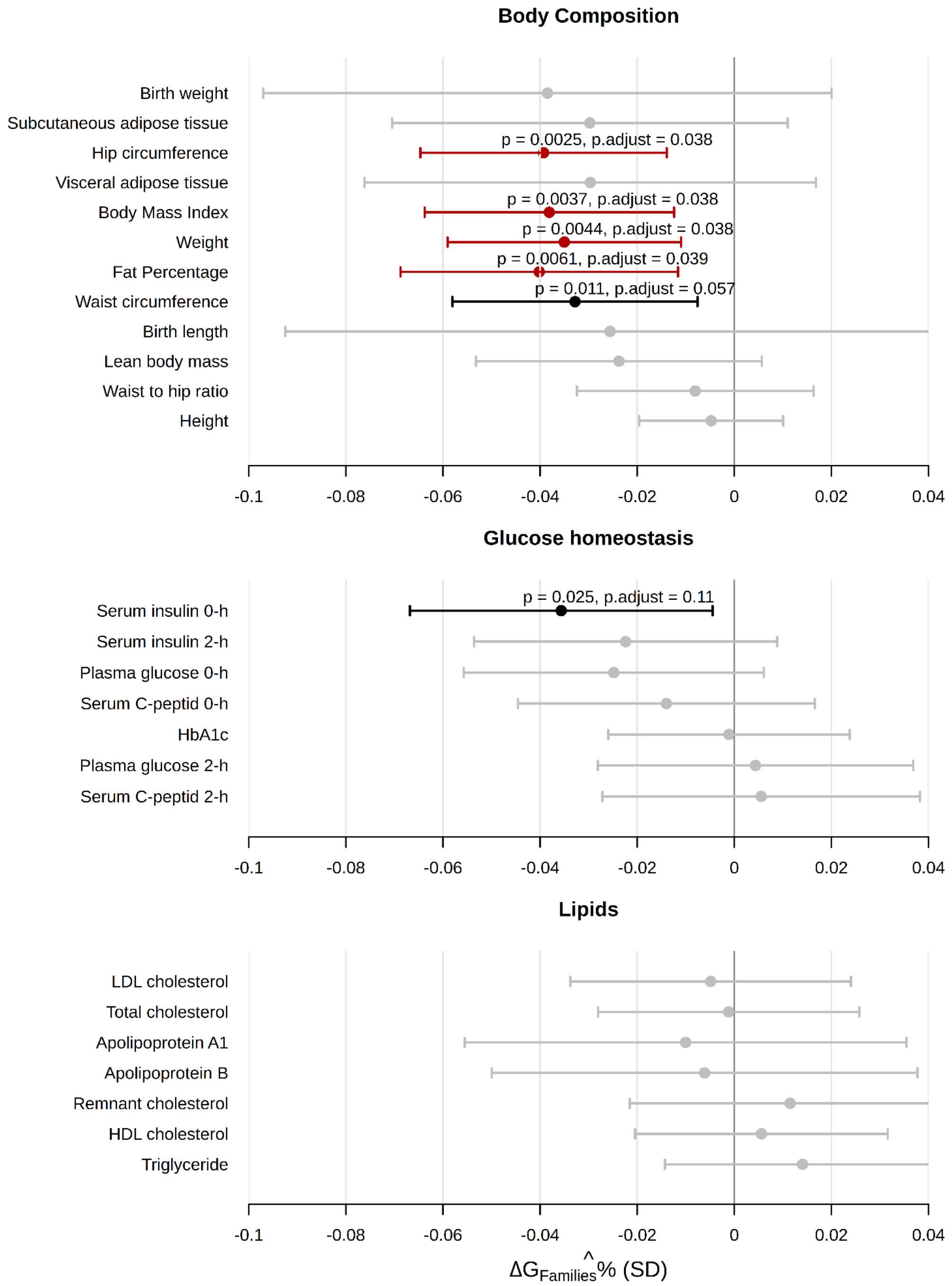

Figure S4. Estimated difference in mean genotypic values by model 2. Estimates of differences in mean genotypic value, $\Delta\widehat{G_{Families}}$, obtained from admixed full siblings with a more complex model that takes into account that some of the siblings in the dataset are from the same families (using model 2). Points show the estimates for transformed phenotypes using age and sex as covariates. Error bars are the 95% confidence intervals. Red colour means FDR adjusted p value < 0.05.

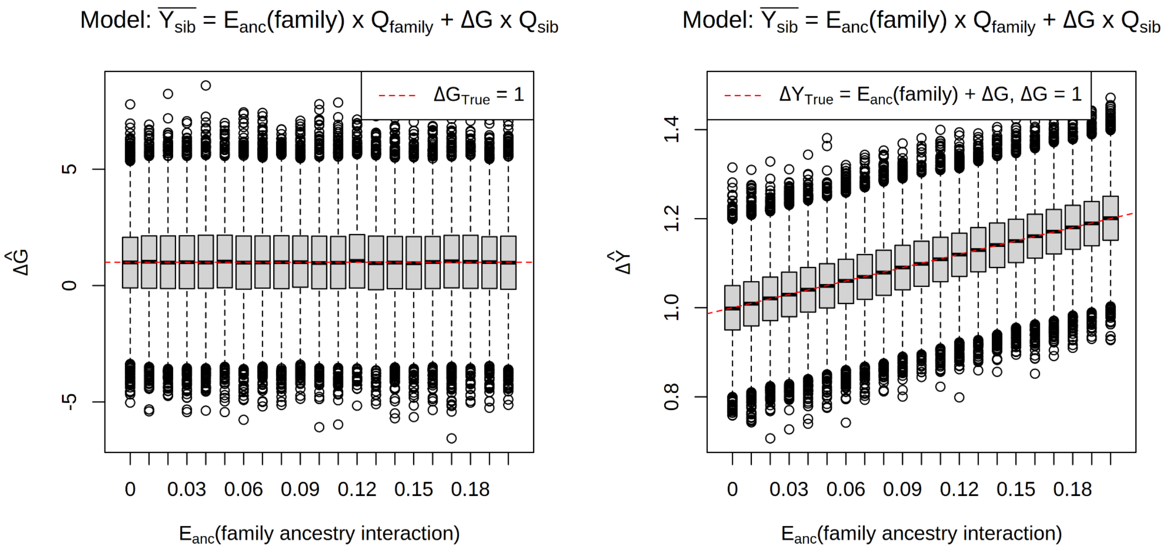

Figure S5. Estimation of ΔG and ΔY with simulated ancestry-by-environment interaction upon each family's average ancestry proportion. Results from a simulation of ancestry-by-environment interaction where the environment is correlated with the family's average ancestry proportion and each sibling's phenotype value is as $y_{ij} \sim N(Q_{ij}\Delta G + E_{anc}\overline{Q_j}, \sigma^2)$. The simulations were based on the sibling pairs from this study and their admixture proportions. The left plot shows the estimated difference in mean genotypic values using model 1b, and the right plot shows the estimated difference in mean phenotypic values using 1a. We performed 10,000 simulations for each value of E_{anc}.

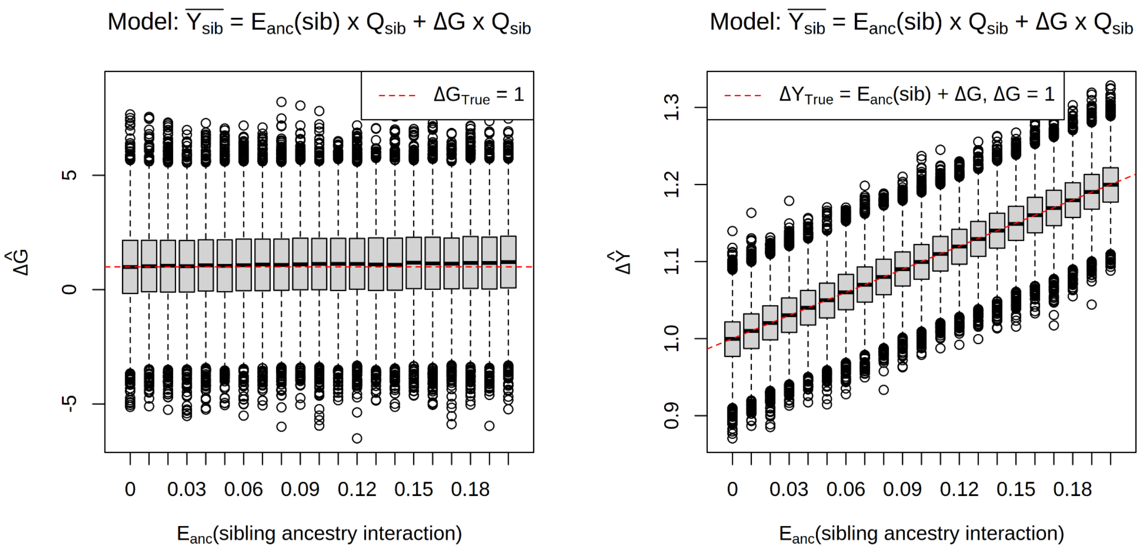

Figure S6. Estimation of ΔG and ΔY with simulated ancestry-by-environment interaction upon each sibling's ancestry proportion. Results from a simulation of ancestry-by-environment interaction where the environment is correlated with the individual sibling's ancestry proportion and each sibling's phenotype value is as $y_{ij} \sim N(Q_{ij}\Delta G + E_{anc}Q_{ij}, \sigma^2)$. The simulations were based on the sibling pairs from this study and their admixture proportions. The left plot shows the estimated difference in mean genotypic values using model 1b, and the right plot shows the estimated difference in mean phenotypic values using 1a. We performed 10,000 simulations for each value of E_{anc}.

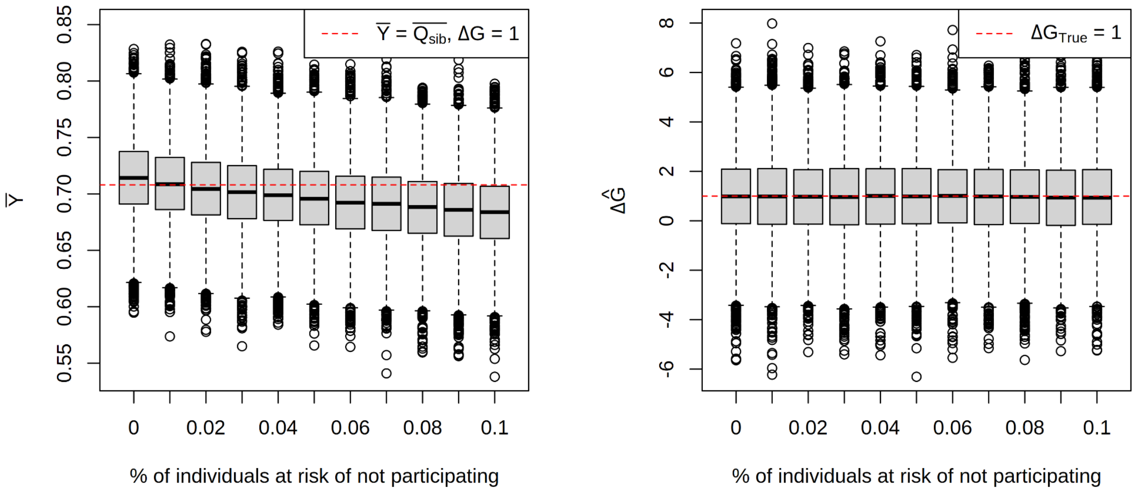

Figure S7. Estimation of ΔG and ΔY with simulated participation bias based on siblings' phenotypic values.

Results from a simulation of participation bias where individuals with a "high" phenotype value have a high (20%) probability of not participating in the study. The left plot shows the mean phenotypic value across all participating individuals and the right plot shows the estimated difference in mean genotypic values between the populations. In both plots each box corresponds to results from a specific threshold for defining a "high" phenotype value based on the top quantiles of the data (ranging from 0 to 10%). If one individual does not participate then their sibling will also be removed. The simulations were based on the sibling pairs from this study and their admixture proportions. To keep the number of individuals the same regardless of degree of participation bias a new phenotype was simulated for non-participating siblings.

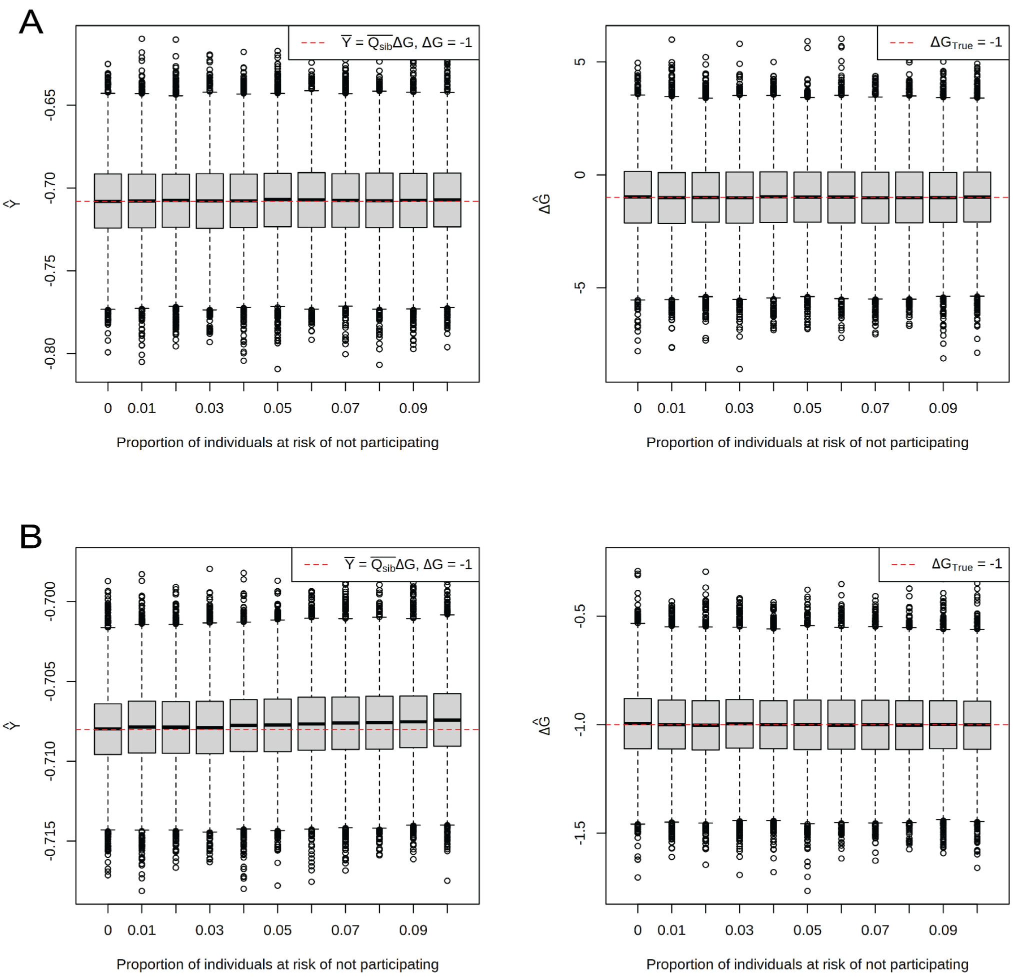

Figure S8. Estimation of ΔG and ΔY with simulated participation bias based on mothers' phenotypic values.

Simulations of participation bias. We define siblings at risk of not participating if their mother has a high Inuit ancestry proportion above 0.8 and a low phenotype value. Siblings at risk have a 20% probability of not participating in the study. Both mothers and their offspring were then simulated to have phenotype values following $N(Q_{ij}\Delta G, \sigma^2)$. σ^2 = 1 in panel A, σ^2 = 1/10 in panel B. We have estimated the ancestry proportion for both parents, however, we don't know which one is the mother. We chose a random parent as the mother. We defined the threshold of having a low phenotype based on the quantiles of the simulated phenotypes of the mothers and performed simulations for different quantiles. We performed 10,000 simulations for each threshold and estimated ΔG using model 1b and calculated the mean phenotype value of all participating siblings as $\hat{Y}$.

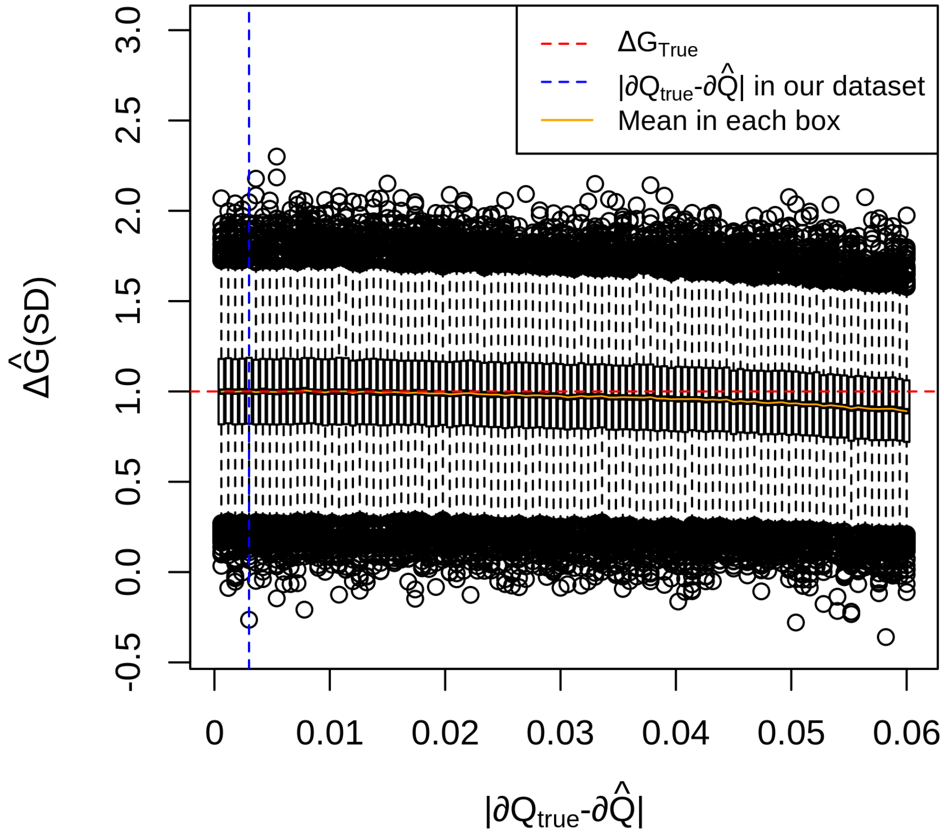

Figure S9. Bias in the estimation caused by error in ancestry inference estimated using simulations. Each box shows the estimates of $\widehat{\Delta G}$ measured in SD for a given absolute mean error size in ∂Q with the middle line showing the median and the box indicating the 25th and 75th quantiles. We used ∂Q of siblings in our study as the true ∂Q. The orange line shows the mean of the same estimates. The red dotted line shows the true difference in genotypic values that was simulated and the blue dotted line shows an estimate of the ∂Q present in the data analysed in this study based on F1 siblings. All the estimates were performed with model 1b.
